# Social skills in neurodevelopmental disorders: a study using role-plays to assess adolescents and young adults with 22q11.2 deletion syndrome and autism spectrum disorders

**DOI:** 10.1186/s11689-024-09527-y

**Published:** 2024-03-18

**Authors:** Clémence Feller, Laura Ilen, Stephan Eliez, Maude Schneider

**Affiliations:** 1 Department of Psychology and Educational Sciences, Clinical Psychology Unit for Intellectual and Developmental Disabilities, Faculty of Psychology and Educational Sciences, University of Geneva, 40, Boulevard du Pont-d’Arve, 1205 Geneva, Switzerland; 2 Developmental Imaging and Psychopathology Lab Research Unit, Faculty of Medicine, University of Geneva, Geneva, Switzerland; 3 Department of Genetic Medicine and Development, Faculty of Medicine, University of Geneva, Geneva, Switzerland

**Keywords:** Social skills, Role-plays, Direct observation, Autism spectrum disorders, 22q11.2 deletion syndrome

## Abstract

**Backgrounds:**

Social skills are frequently impaired in neurodevelopmental disorders and genetic conditions, including 22q11.2 deletion syndrome (22q11DS) and autism spectrum disorders (ASD). Although often assessed with questionnaires, direct assessment provides a more valid estimate of the constructs. Role-plays (i.e., simulates situational settings) therefore appear to be an appropriate indicator of social skills in daily life.

**Methods:**

This co-registered study involved 53 individuals with 22q11DS, 34 individuals with ASD, and 64 typically developing (TD) peers aged 12–30 years. All participants were assessed with role-plays as well as parent-reported questionnaires and clinical interviews focusing on social skills, functioning and anxiety.

**Results:**

Both clinical groups showed impaired social skills compared to TD, but distinct social profiles emerged between the groups. Individuals with 22q11DS displayed higher social appropriateness and clarity of speech but weaker general argumentation and negotiation skills, with the opposite pattern observed in participants with ASD. No association was found between social skills measured by direct observation and caregiver reports. Social anxiety, although higher in clinical groups than in TD, was not associated with role-plays.

**Conclusions:**

This study highlights the need to train social skills through tailored interventions to target the specific difficulties of each clinical population. It also highlights the importance of combining measures as they do not necessarily provide the same outcome.

**Supplementary Information:**

The online version contains supplementary material available at 10.1186/s11689-024-09527-y.

## Introduction

Adolescence is a critical period for socialization, particularly because social expectations increase with age. Indeed, the challenges of individuation and independence take place during this phase, leading youths to adapt their positions and roles in relation to their parents and peers [1]. In addition, the mounting importance of peer interactions and the growing complexity of social relationships appear to be crucial to adolescents outcome (e.g., [2]). Indeed, it was observed in the general population that social difficulties can contribute to lower self-esteem and academic difficulties later in life [3], with social withdrawal and social anxiety being closely interrelated [4, 5]. To navigate successfully in the social sphere and effectively decode the social environment, subtle abilities are required. On this aspect, individuals with neurodevelopmental disorders and certain genetic conditions are penalized. Indeed, social impairments are reported in many of these disorders and conditions, including autism spectrum disorders (ASD) and 22q11.2 deletion syndrome (22q11DS). Both individuals with 22q11DS (e.g., [6, 7]) and with ASD (e.g., [8]) are characterized by social impairments, including difficulties perceiving and interpreting social signals (*i.e.* social skills; [9]), that may interfere with their ability to create, maintain and end social interactions [10]. According to some theoretical models (e.g., [11]), social skills also partially depend on executive, cognitive and verbal competences, all of which are frequently impaired in neurodevelopmental disorders [12, 13].

In ASD, alterations in social communication and social interactions are observed, in addition to repetitive and restrictive behaviors and interests. Several social skills are impaired from very early on, notably social smiling, looking at faces, responding to one’s name and making eye contact [14]. Moreover, individuals with ASD exhibit weaker adaptive behaviors – defined as the skills required to function and be independent in everyday environments [15] – in the social domain. Specifically, individuals with ASD have lower skills in the area of socialization, resulting in difficulties making friends, acting in an appropriate way with peers, etc. [16, 17]. In 22q11DS, a neurogenetic condition affecting approximately 1 in 2000–4000 births [18], social impairments are typically observed in combination with the typical physical (*e.g.*, chronic infections, cleft palate, heart defects, hypocalcemia), cognitive (*e.g.*, IQ around 70, executive functions’ deficits) and psychiatric (*e.g.*, psychosis, attention deficit, anxiety and mood disorders) characteristics of the syndrome [19–21]. Studies have highlighted that individuals with 22q11DS exhibit poorer social functioning compared to typically developing peers (TD). For example, poorer social skills [22] and more problematic social behaviors [23] have been reported in children with 22q11DS compared to their siblings. Moreover, social immaturity and difficulties initiating social interactions [24] as well as socio-communicative impairments [25] have been highlighted. Emotion processing has also been found to be impaired, with difficulties in emotion recognition and an abnormal visual exploration of faces (e.g., [26, 27]). Finally, individuals with 22q11DS exhibit weaker adaptive skills [20] and are described as socially withdrawn and more socially inhibited and isolated than their peers (e.g., [28]). Of note, social skills interventions remain scarce in 22q11DS, but some studies have reported positive outcomes (e.g., [29, 30]).

Social skills thus appear to be of critical importance to better characterize the social profile of each of these conditions, as differences in the social phenotype of 22q11DS and idiopathic ASD have been highlighted. Indeed, differences were observed in socio-emotional reciprocity, idiosyncratic speech and non-verbal interactions [27, 31, 32]. Moreover, higher levels of empathy, sense of humor and other complex social skills were found in individuals with 22q11DS compared to youth with idiopathic ASD [33]. In contrast, some authors have suggested that a significant proportion of 22q11DS meet criteria for ASD (e.g., [34, 35]) and reported similarities in emotion recognition, conversations initiation and maintenance, and adaptive socialization [27, 31, 32]. A precise characterization of their unique social skills profile would enable to tailor clinical interventions, such as social skills training (e.g., [36–39]), to the individual needs of each population. Finally, the link between social skills and social anxiety—a frequent comorbidity of both ASD and 22q11DS [20, 40]—requires further exploration, since previous studies reported a negative association between these two dimensions [41, 42].

An important limitation in the field is that social skills are typically assessed through questionnaires, which represents the most classical method to examine this construct [43]. Nevertheless, most of the existing questionnaires have been developed for a typical population. As a result, they are often complex and lengthy, and are also dependent on the awareness of difficulties that is frequently absent in clinical populations [44]. Moreover, some questionnaires are completed by caregivers, introducing another perspective that does not necessarily correspond to direct observation [45]. Indeed, direct observation is assumed to provide a more valid estimate than more distal measures such as questionnaires [46]. To assess social skills in the most ecological way, it is necessary to approximate a person’s behavior in different daily-life situations, which can be done using role-plays. These systematic and direct observations of social behaviors are considered the *gold standard* of social skills assessment, as they provide access to real-world behaviors through simulated situational settings [47]. This method has been successfully used in various clinical populations (e.g., [45, 47, 48]), including youth with ASD [49, 50]. For instance, the Social Skills Performance Assessment (SSPA; [51]) was initially developed to assess individuals with schizophrenia through role-plays (e.g., [52–54]) but was also used in other conditions (e.g., [55]), including ASD. Indeed, Verhoeven et al. [48] found the SSPA to be an appropriate tool for assessing social skills in a sample of adults with ASD. Ratto et al. [56] developed the Contextual Assessment of Social Skills (CASS) that was specifically designed to measure social skills in individuals with ASD. This task was found to successfully discriminate between ASD and non-ASD individuals and was then used in several studies to assess treatment outcome after a social skills training (e.g., [57, 58]). However, it should be noted that role-playing games, although approximating reality, are performed in a laboratory setting that remains somewhat artificial [59]. Moreover, a paucity of studies has examined the convergent validity between self-reported measures and role-plays. For instance, Verhoeven et al. [48] found no relationship between the SSPA and self-reported measures of social functioning, indicating low convergent validity. This suggests that these methods explore different aspects of the same construct, in this case social skills. It is also important to note that these role plays measures have been developed to characterize social skills but are not diagnostic instruments, unlike standardized assessments such as the Autism Diagnostic Observation Schedule [60] or the Brief Observation Of Symptoms of Autism (BOSA; [61]) that are also based on direct observation. Unlike role plays, they require extensive training and are primarily used to identify ASD-related manifestations *at large* and not just social skills. In summary, although direct observation is increasingly used in neurodevelopmental disorders, its purpose may differ depending on whether it is used for diagnostic assessment or to characterize the social skills profile.

### Aims of the study

The first aim of the present study was to investigate social skills using semi-standardized role-plays (*i.e.* the Social Skills performance Assessment (SSPA; [51])) in two neurodevelopmental disorders often considered to share similar social profiles and to examine the correspondence with a more standard methodology (*i.e.* parent-report). The second aim was to investigate the potential association between social skills and social anxiety. First, participants with ASD and 22q11DS were expected to report lower social skills compared to TD, as measured both by direct observation and through parental report, and to observe a significant association between the two measures. We also expected to observe different patterns of social skills in the clinical groups, as well as significant associations between social skills and social functioning. Second, compared to TD, higher levels of social anxiety were expected in both clinical groups, with higher social anxiety being associated with lower social skills (as measured by direct observation but also by caregiver report).

## Methods

### Participants

One hundred and fifty-one participants (47% female) aged 12 to 30 years were included in the study (mean age = 18.77, SD = 4.39). Thirty-four (44% female) individuals with ASD (mean age = 19.97, SD = 5.03) were recruited from clinical centers in Geneva and France, through a network of medical professionals and through announcements to family associations in Switzerland and France. Fifty-three (43% female) 22q11DS carriers (mean age = 19.31, SD = 4.62) were recruited through the 22q11DS Swiss longitudinal cohort which includes both Swiss and French individuals. Sixty-four (52% female) individuals were in the TD group (mean age = 18.76, SD = 3.82) and were recruited through siblings of 22q11DS carriers and through announcements at the University of Geneva. Written consent was requested from caregivers for all participants with ASD and 22q11DS, as well as for TD under 18 years. This study was approved by the Swiss Ethics Committee on research involving humans (Commission Cantonale d’Ethique de la Recherche sur l’Etre Humain – CCER) in Geneva (CH).

Inclusion criteria for all participants were 1) age between 12 and 30 years and 2) sufficient command of the French language. All participants from the ASD group had a confirmed clinical diagnosis of ASD. They were assessed using the Autism Diagnostic Observation Schedule, second version (ADOS;[60], and their caregivers using the Autism Diagnostic Interview-Revised (ADI-R; [62]) or the Social Communication Questionnaire (SCQ; [63]). All participants in the 22q11DS group had a confirmed genetic diagnosis of microdeletion 22q11.2 (determined by fluorescence in situ hybridization, multiplex ligation-dependent probe amplification, or micro-array analysis). They were assessed with the Social Communication Questionnaire (SCQ; [63]) with a mean score of 11.25. Individuals with ASD and 22q11DS were screened for comorbid psychiatric disorders using a validated semi-structured instrument: Diagnostic Interview for Children and Adolescents-Revised (DICA [64]; or Schedule for Affective Disorders and Schizophrenia for School-Age Children Present and Lifetime Version (K-SADS-PL DSM-5; [65] for participants under 18 years old and Structured Clinical Interview for DSM-IV Axis I (SCID-I; First & Williams, 1996 or DSM-5 (SCID-5-CV; First, Williams, & Spitzer, 2016) for participants above 18 years old. Comorbidities, medication and ASD scores are displayed in Table 1. Note that all participants were assessed using the Wechsler Intelligence Scales for Children or Adults (WISC-V [66]; or WAIS-IV [67];) but intellectual deficiency was not an exclusion criterion. For TD, exclusion criteria were 1) being born preterm, 2) having a first-degree relative with any developmental disorder (siblings of participants with 22q11DS were included if the 22q11.2 deletion was confirmed as de novo), 3) having a lifetime history of psychiatric (including neurodevelopmental disorders such as ASD), neurological, or learning disorders. Of note, TD were also assessed with the SCQ, with a mean score of 2.94 and none of the participants scoring above the clinical cutoff. Sample characteristics are displayed in Table 1.

**Table 1:** Participant characteristics, psychiatric diagnoses and psychotropic medication

		Diagnostic group		
		TD	22q11 DS	ASD
*N*		64	53	34
Gender (female (%))		33 (52%)	23 (43%)	15 (44%)
Age (mean (SD))		18.76 (3.82)	19.31 (4.62)	19.97 (5.03)
Full Scale IQ (mean (SD))		111.25 (12.54)	71.38 (14.04)	104.59 (17.322)
Psychiatric diagnoses (*N*(%))	Simple phobia		9 (17%)	6 (18%)
	Agoraphobia		0	4 (12%)
	Social phobia		1 (2%)	6 (18%)
	Generalized anxiety		13 (25%)	7 (21%)
	Attention deficit disorder		23 (44%)	10 (29%)
	Mood disorders		5 (9%)	14 (41%)
	Psychotic disorders		3 (6%)	0
	Oppositional defiant disorder		1 (2%)	1 (3%)
	Obsessive–compulsive disorder		2 (4%)	2 (6%)
	Others		2 (4%)	1 (3%)
ADOS-2 module 3 (*N* = 10) (mean)	ADOS-2 total score			11.7
	ADOS-2 SA score			8.1
	ADOS-2 RRB score			3.6
ADOS-2 module 4 (*N* = 24) (mean)	ADOS-2 total score			12.37
	ADOS-2 SA score			9.29
	ADOS-2 RRB score			3.08
ADI-R (*N* = 21) (mean)	ADI-R domain A			17.76
	ADI-R domain B			13.04
	ADI-R domain C			5.76
Questionnaires/interviews (mean (SD))	Vineland (socialisation score)	49.96 (4.88)	43.15 (27.30)	31.26 (5.65)
	ERSSQ total score	85.56 (15.40)	55.47 (15.92)	49.27 (14.56)
	SIAS total score	22.46 (14.49)	31.08 (16.47)	46.80 (16.27)
	SCQ total score	2.94 (2.84)	11.25 (6.87)	17.47 (8.13)
Psychotropic medication	Total (*N*(%))		31 (59%)	11 (33%)
Categories	Psychostimulant		14 (26%)	3 (9%)
	Antidepressants		13 (24%)	3 (9%)
	Neuroleptics		12 (22%)	3 (9%)
	Antiepileptics		3 (6%)	0
	Anxiolytics		4 (8%)	2 (6%)

TD have been screened for psychiatric diagnostics conforming to our exclusion criterias

Mood disorders include depressive disorder, dysthymia, bipolar disorder and mood dysregulation

Psychotic disorders include schizoaffective disorder and schizophrenia

Others include gambling disorders and enuresia

Medication: total = number of participants under medication (some participants take more than one medication)

### Material

#### Social skills

All participants were assessed with an adapted version of the Social Skills Performance Assessment (SSPA; [51]). Participants were first given a 1-min practice role-play to familiarize with the task. In this role-play, they had to decide what to do after school/work with their friend, played by the examiner. They were then asked to perform two fictitious situations of 3 min each: meeting a new neighbor and retrieving a notebook loaned to a classmate. For each situation, examiners followed predetermined settings with detailed instructions that included specific prompts. The role-plays were videotaped for double scoring. CF and MS updated the scoring descriptions of each category and agreed on several videos before training other examiners. They were then asked to score videos by recording their rational for each score. If they disagreed on a grade, they discussed it with arguments and agreed together on the final grade. Of note, 96% of the videos were double rated, with the missing 4% due to technical problems during recording. In the original paper, the intraclass correlation coefficient (ICC) between raters was of 0.91 [51], and in the validation study in an ASD sample, validation coefficients were of 0.80 for the ICC and of 0.86 for the Cronbach’s a [68]. For role-plays 1 and 2, coded behaviors included: involvement (interest/disinterest in the conversation), non-verbal communication and affect (eye gaze, voice tone, etc.), social adequacy (appropriateness), fluency (ease of speech production), clarity (comprehensibility of the speech), and focus (ability to stay in role-plays). In role-play 1, there was an additional code of overall conversation (a global measure of the ability to interact and establish a relationship). In role-play 2, there were three additional codes: submission/persistence (insistence of the participant to retrieve the notebook despite the examiner refusal), negotiation ability (solutions brought by the participant to solve the problem) and overall argument (balance between the degree to which the participant played a part in the argument and the degree to which he needed help from the examiner). Of note, subscales were found to be highly related to each other in a network study conducted by Hasson-Ohayon et al. [55] that also reveals that the most central items were social appropriateness, focus and clarity. Each subscale is rated on a 5-points Likert scale, with seven subscales for the first role-play and nine for the second one. For each category, short descriptions are provided in a coding booklet, along with examples. The descriptions are not exhaustive but give references for the scoring. Higher scores indicate more effective social skills. Further details about the scoring are available upon request.

All the caregivers completed the Emotion Regulation and Social Skills Questionnaire (ERSSQ; [69]) containing 27 items answered on a 5-point Likert scale (never, rarely, sometimes, often, always). Higher scores indicate better social skills. There are two versions of this questionnaire, one from individuals under 18 years old and one from individuals above 18 years old. The versions are very similar except for some items (items 4, 7, 16 and 17) that are adapted to the context (i.e., work or school). Seventy-six participants were evaluated with the adolescent version (TD *n* = 29, 22q11DS *n* = 25, SAD *n* = 22) and sixty-seven with the adult version (TD *n* = 28, 22q11DS *n* = 28, ASD* n* = 11). Mean values are displayed in Table 1.

#### Social functioning

The Vineland Adaptive Behavior Scale, 2nd Edition (VABS-II; [15]) was administered to the parents when the participants were still living at home (*N* = 116; TD = 42, ASD = 27, 22q11DS = 47) to assess adaptive functioning. Only the socialization dimension was used in the analyses using appropriate standardized scores (M = 100; SD = 15). Mean values are displayed in Table 1.

#### Social anxiety

All participants were assessed with the Social Interaction Anxiety Scale (SIAS; [70]), a self-reported questionnaire assessing social anxiety with 20 statements answered on a 5-point Likert scale (not at all, slightly, moderately, very, extremely). Higher scores indicate greater social anxiety. Mean values are displayed in Table 1.

### Statistical Analysis

Statistical analyses were performed with IBM SPSS Statistics 26. Non-parametric statistics (Kruskal–Wallis tests and Spearman correlations) were performed because the distribution of our variables of interest did not follow a normal distribution (Shapiro–Wilk tests *p* < 0.05). For post-hoc analyses, only adjusted *p* values are reported to account for Bonferroni multiple testing correction. Note that that for correlations, we controlled for gender, age and IQ. Regarding the first aim, group comparisons were conducted on social skills measured with role-plays (i.e., SSPA total score and subscales; group comparisons were also conducted on role-play 1 and 2 separately to examine the consistency of the results across contexts) and through parent report (i.e. ERSSQ). Secondly, and similarly to what has been done by Morrisson et al. (2017), a discriminant function analysis (DFA) was conducted as a *post-hoc* analysis to determine the constellation of social skills that best characterized group membership to further explore specific profiles. Thirdly, correlations were conducted between directly observed social skills (i.e., SSPA) and parent-reported social skills (i.e., ERSSQ) in each clinical group, as well as between social skills (both SSPA and ERSSQ) and social functioning (i.e. Vineland Socialization Standard Score). Regarding the second aim, the social anxiety level (i.e., SIAS) was compared across groups, and correlations were conducted between social anxiety (i.e., SIAS) and social skills (both SSPA and ERSSQ). Finally, *post-hoc* correlations between socials skills (both SSPA and ERSSQ) and general characteristics of the participants (intellectual quotient (IQ), age, gender, and ASD symptomatology (in participants with ASD only)) were conducted and are presented in supplementary material. Of note, this study was co-registered (10.17605/OSF.IO/QF6WN), and only the post-hoc correlations deviate from the original statistical analysis plan.

## Results

### Sample characteristics

Participants were not statistically different in terms of age and gender (all *p* > 0.05). However, participants differed in terms of IQ (*H*(2) = 86.955, *p* < 0.001). Pairwise comparisons showed that participants with 22q11DS had significantly lower full-scale IQ score than both participants with ASD (*p* < 0.001) and TD (*p* < 0.001). Mean values are displayed in Table 1. Of note, two participants with ASD and twenty-one participants with 22q11DS had an IQ score in the intellectual disability range (IQ < 70). All the analyses were replicated without the participants with 22q11DS (*n* = 14) who scored above the clinical cutoff (15) on the SCQ questionnaire and results remained unchanged (data not shown). We also compared 22q11DS participants scoring above and below the SCQ clinical cutoff and found no statistically significant differences (see Supplementary Material).

### Aim 1: Social skills in neurodevelopmental disorders

#### a. Direct observation (i.e. SSPA): group differences and specific profiles

Statistically significant differences were observed among the groups on social skills, as measured with the SSPA (*H*(2) = 80.644, *p* < 0.001, η^2^ = 0.531). *Post-hoc* analysis showed that TD participants showed higher social skills than both participants with 22q11DS (*H*(2) = 61.221, *p* < 0.001, η^2^ = 0.524) and with ASD (*H*(2) = 69.101, *p* < 0.001, η^2^ = 0.709). There was no difference between the clinical groups (*p* > 0.05). When adding gender, age and IQ as covariates, the group differences between TD participants and the clinical groups remained significant and a significant difference emerged between participants with ASD and those with 22q11DS, the latest showing greater social skills. See Supplementary Material. Similar results were found when analyzing role-play 1 and 2 individually. See Supplementary Material. Note that the total scores for the two role-plays were strongly associated (r = 0.779, *p* < 0.001).

When looking at each subscale constituting the SSPA total score, statistically significant differences were observed across groups, with higher scores for TD participants compared to both participants with 22q11DS and with ASD for all the subscales. See Table 2 for detailed results. Participants with ASD and those with 22q11DS statistically differed from each other on two subscales: participants with ASD scored lower on the “social adequacy” subscale (*H*(2) = 28.661, *p* = 0.007, η^2^ = 0.325) but higher on the “assertiveness (persistence/submission)” subscale (*H*(2) = -18.632, *p* = 0.039, η^2^ = 0.231) than participants with 22q11DS. However, the latest result did not survive Bonferroni correction. When adding gender, age and IQ as covariates, participants with 22q11DS performed significantly better on many subscales compared to participants with ASD (non-verbal communication, clarity, fluency, involvement, social adequacy and conversation). See Supplementary Material for detailed results.

**Table 2:** SSPA subscales performance

	Performance mean % (SD)			Groups comparisons			Post-Hoc analyses								
							TD-22q11DS			TD-ASD			22q11DS-ASD		
	TD	22q11DS	ASD	Kruskal–Wallis test	*p*-value	η^2^	Test statistic	*p*-value	adjusted *p*-value	Test statistic	*p*-value	adjusted *p*-value	Test statistic	*p*-value	adjusted *p*-value
SSPA total non-verbal communication and affect (RP 1 + 2)	8.84 (1.37)	6.70 (1.96)	6.29 (1.69)	56.564	**.000**	0.369	48.940	.000	**.000**	59.028	.000	**.000**	10.087	.287	.861
SSPA total clarity (RP 1 + 2)	9.62 (.68)	8.42 (1.25)	7.44 (2.06)	48.855	**.000**	0.317	40.641	.000	**.000**	55.761	.000	**.000**	15.120	.098	.295
SSPA total fluency (RP 1 + 2)	9.41 (.97)	7.40 (1.52)	6.91 (2.08)	61.722	**.000**	0.404	53.094	.000	**.000**	58.138	.000	**.000**	5.044	.591	1.000
SSPA total focus (RP 1 + 2)	9.28 (1.16)	8.25 (1.52)	8.15 (2.07)	21.609	**.000**	0.132	33.995	.000	**.000**	28.059	.002	**.005**	-5.936	.518	1.000
SSPA total involvement (RP 1 + 2)	8.84 (1.12)	7.32 (1.52)	7.15 (1.79)	40.114	**.000**	0.258	43.719	.000	**.000**	46.072	.000	**.000**	2.353	.250	1.000
SSPA total social adequacy (RP 1 + 2)	8.83 (1.13)	8.06 (1.66)	6.94 (1.68)	29.940	**.000**	0.189	20.813	.009	**.026**	49.475	.000	**.000**	28.661	.002	**.007**
SSPA overall conversation (RP 1)	4.34 (.88)	3.21 (.948)	2.82 (.716)	58.865	**.000**	0.384	45.610	.000	**.000**	61.409	.000	**.000**	15.800	.097	.262
SSPA overall argument (RP 2)	4.23 (.729)	3.04 (1.96)	3.27 (1.13)	46.061	**.000**	0.298	50.286	.000	**.000**	38.805	.000	**.000**	-11.481	.214	.642
SSPA negociation ability (RP 2)	4.23 (.88)	3.11 (.87)	3.36 (1.05)	37.702	**.000**	0.241	45.930	.000	**.000**	34.578	.000	**.000**	-11.352	.221	.664
SSPA submission/persistence (RP 2)	4.58 (.66)	3.62 (.90)	3.85 (1.37)	31.743	**.000**	0.201	42.442	.000	**.000**	23.810	.006	**.019**	-18.632	**.039**	.118

Significant *p*-values at the 0.05 level are displayed in bold

Adjusted *p*-value consider Bonferonni correction for multiple comparison tests

*RP* stands for role-plays, *SD* stands for standard deviation, *TD* stands for typically developping peers, *22q11DS* stands for participants with microdeletion 22q11.2, *ASD* stands for participants with autism spectrum disorders

**Fig. 1:**
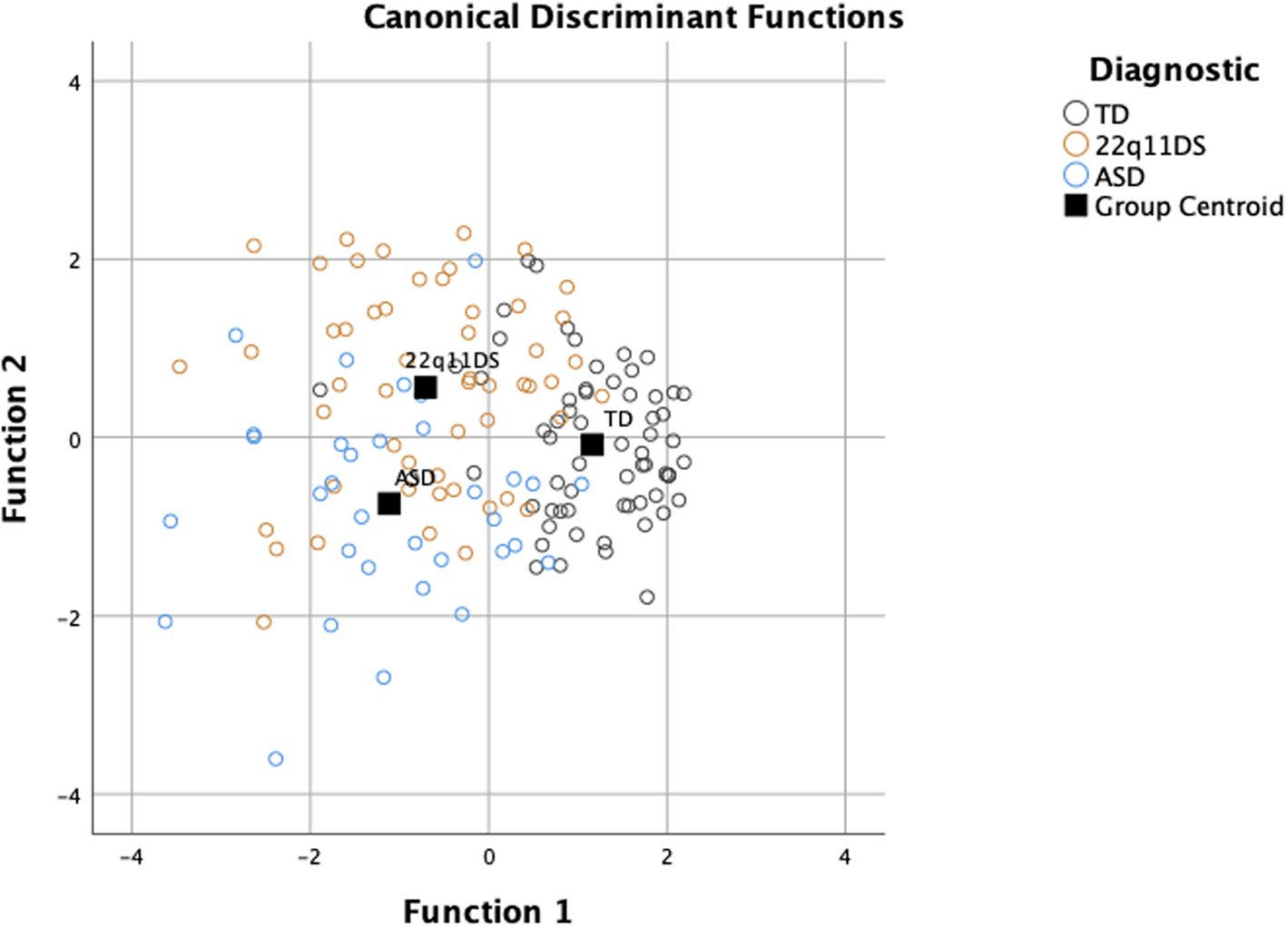
Canonical Discriminant Functions Legend: Discriminant Function Plot. Group centroids (squares; mean linear combinations) and the linear combination for each participant (circles) are plotted. Two functions separate groups: Function 1 (x-axis) best separates TD from ASD and 22q11DS, and Function 2 (y-axis) separates ASD from 22q11DS

The DFA resulted in one function separating the TD group from the ASD and 22q11DS groups that accounted for 81.4% of the variance (Wilk’s = 0.394, k(20) = 132.65, *p* < 0.001, canonical R^2^ = 0.715). A second function was also significant, separating the ASD from the 22q11DS group (Wilk’s = 0.807, k(9) = 30.54, *p* < 0.001, canonical R^2^ = 0.439). Figure 1 displays the DFA plot. The pattern of standardized coefficients (see Table 3) indicates that TD participants are best differentiated from the two clinical groups by better overall conversational skills, whereas participants with ASD and 22q11DS are characterized by differences regarding more specific conversational skills, with higher social adequacy and clarity and lower overall argument and negotiation abilities for 22q11DS participants, the reverse pattern being observed in ASD participants.

**Table 3:** Standardized Coefficients for SSPA subscales for Discriminant Functions 1 and 2

	Function	
	1	2
SSPA total social adequacy (RP 1 + 2)	.086	**.631**
SSPA total non-verbal communication and affect (RP 1 + 2)	.137	-.324
SSPA overall argument (RP 2)	.153	**-.559**
SSPA total clarity (RP 1 + 2)	.236	**.720**
SSPA overall conversation (RP 1)	**.519**	**.480**
SSPA total fluency (RP 1 + 2)	.371	-.166
SSPA total involvement (RP 1 + 2)	-.252	-.185
SSPA overall argument (RP 2)	-.238	-.200
SSPA negociation ability (RP 2)	.217	**-.493**
SSPA submission/persistence (RP 2)	.195	.123

Coefficients greater than .40, shown in bold, load onto the function

*TD* stands for typically developping peers, *22q11DS* stands for participants with microdeletion 22q11.2, *ASD* stands for participants with autism spectrum disorders, *SD* stands for standard deviation

#### b. Parent-reported social skills (i.e. ERSSQ): group differences

Statistically significant differences were also observed among the groups on social skills, as measured with the ERSSQ (*H*(2) = 74.241, *p* < 0.001, η^2^ = 0.488). Post-hoc analysis showed that TD participants showed higher social skills than both participants with 22q11DS (*H*(2) = 55.624, *p* < 0.001, η^2^ = 0.475) and with ASD (*H*(2) = 67.652, *p* < 0.001, η^2^ = 0.694). There was no difference between the clinical groups (*p* > 0.05).

#### c. Association between direct observation (i.e. SSPA) and other-reported social skills (i.e. ERSSQ)

The SSPA total score was not significantly associated with the ERSSQ total score in the 22q11DS group (*r* = 0.229, *p* = 0.113) or across participants with ASD (*r* = -0.074, *p* = 0.696) but the associations was significant in TD (*r* = 0.340, *p* = 0.015).

#### d. Social skills (i.e., SSPA and ERSSQ) and social functioning (i.e. VABS)

The VABS socialization domain was not significantly associated with the SSPA total score in any of the three groups (all *p* > 0.05). However, it was robustly associated with the ERSSQ total score in both clinical groups (all *p* < 0.001) but not in TD (*r* = 254, *p* = 0.104).

### Aim 2: Does social anxiety play a role in social skills?

#### a. Group differences on social anxiety (i.e. SIAS)

Statistically significant group differences were observed on the SIAS total score (*H*(2) = 22.019, *p* < 0.001, η^2^ = 0.135). *Post-hoc* analyses showed that TD participants reported lower social anxiety than both participants with 22q11DS (*H*(2) = -12.712, *p* = 0.018, η^2^ = 0.119) and with ASD (*H*(2) = -45.974, *p* < 0.001, η^2^ = 0.489). Moreover, participants with 22q11DS showed lower social anxiety than participants with ASD (*H*(2) = -27.262, *p* = 0.024,, η^2^ = 0.332).

#### b. Social anxiety (i.e. SIAS) and social skills (i.e., SSPA and ERSSQ)

**Fig. 2:**
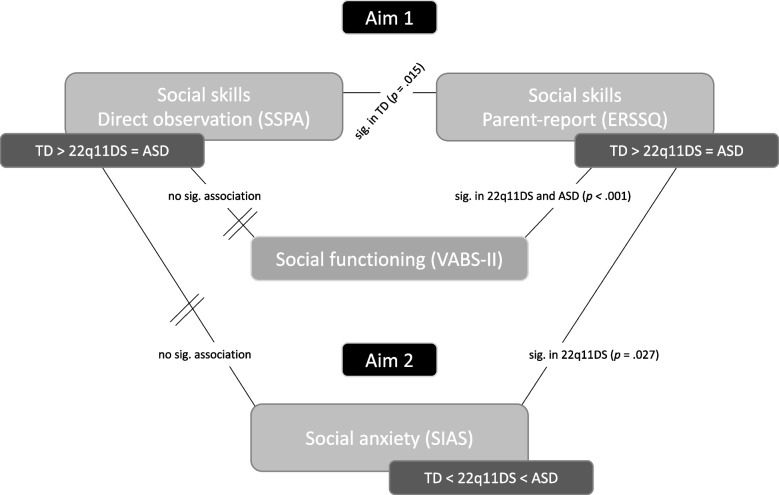
Visual summary of the main results

There was no association between the SSPA total score and the SIAS total score in any of the three groups (TD: *r* = 0.159, *p* = 0.280; 22q11DS: *r* = -0.114, *p* = 0.437; ASD: *r* = 0.360, *p* = 0.250). However, the ERSSQ total score was associated with the SIAS total score in 22q11DS participants (*r* = -0.313 *p* = 0.028), but not in TD (*r* = -0.215, *p* = 0.156) nor ASD participants (*r* = -0.120, *p* = 0.711). See Fig. 2 for a visual summary of the results.

## Discussion

The first objective of this study was to better characterize social skills in two neurodevelopmental disorders often considered to share similar social characteristics. In this regard, we also explored the potential correspondence between a direct observation of social skills (i.e., role-plays SSPA) and a parent-report (i.e., ERSSQ). The second objective was to explore the association between social skills and social anxiety. Finally, additional analyses were conducted to investigate the association between social skills and general characteristics (i.e., age, gender, ASD symptomatology) of the participants. Regarding the first aim, our main findings indicate that participants with 22q11DS and with ASD were characterized by lower social skills compared to TD, either when measured through direct observation (i.e., SSPA) or reported by the caregivers (i.e., ERSSQ). However, the two social skills measures (i.e., SSPA and ERSSQ) were not associated with each other. Whereas the two clinical groups had a comparable score on a global measure of social skills (SSPA total score), population-specific challenges emerged: participants with ASD exhibited less socially adapted behaviors during the role-plays, and participants with 22q11DS tend to be less assertive during the social interactions. Better general conversational skills best distinguished TD participants from the two clinical groups, while the ASD and 22q11DS participants differed from each other in more specific conversational skills: participants with 22q11DS displayed higher social appropriateness and clarity of speech but weaker general argumentation and negotiation skills, with the opposite trend observed in ASD participants. Regarding the second aim, although greater social anxiety (i.e., SIAS) was reported in participants with 22q11DS and ASD compared to TD, it was not associated with directly observed social skills (i.e., SSPA) in any of the groups. However, social anxiety (i.e., SIAS) was associated with parent-reported social skills (i.e., ERSSQ) in participants with 22q11DS only.

### Aim 1: social skills in neurodevelopmental disorders

#### Social skills in ASD and 22q11DS

Participants with ASD and 22q11DS exhibited poorer social skills than TD, consistently with previous studies [6, 8, 71]. Indeed, both on the SSPA total score and on specific subscales, participants with ASD and 22q11DS presented greater difficulties during real-world social interactions compared to TD. Parents also reported poorer social skills in the two clinical groups. However, some distinctions emerged between the two clinical groups, implying not only common social impairments but also population-specific challenges. Of note, this difference in profiles was corroborated by the DFA analysis in which the two clinical groups were well discriminated from each other. Moreover, the addition of IQ as a covariate enhanced the differences between the clinical groups, which reinforces the idea of a distinct social skills profile in the two populations that is not driven by IQ differences.

Among individuals with ASD, more difficulties adjusting to the social situation were reported. Indeed, participants with ASD tended to be overly familiar by asking inappropriate questions or being excessively insistent and indelicate. This finding is consistent with a study on social appropriateness judgments in children and adolescents with ASD, which showed that it was harder for participants with ASD to identify inappropriate behaviors as well as to explain why they were inappropriate [72]. In addition, lack of social awareness has been described as part of the ASD phenotype since the very first studies on ASD [73]. Psycho-educational techniques, such as video-feedback interventions, were shown to be a reliable method to help individuals with ASD decode social cues and improve their social skills, such as engaging in conversation or being empathetic and reciprocal. Therefore, these techniques could be used to increase socially appropriate behaviors during social interactions (e.g., [74, 75]). However, there is an ongoing debate regarding how to teach social skills most effectively (e.g., [76]), and whether training social skills may have long-term negative effects, including increased camouflage (e.g., [77]). Recent studies in ASD point toward a different interaction style rather than a deficit in terms of social skills per se (e.g., [78, 79]). Overall, it is important to keep in mind that appropriateness remains subjective and dependent on the person assessing it, which induces variations when the perspective is taken by a neurotypical or from a neuroatypical person [78, 79].

In contrast, participants with 22q11DS displayed more socially appropriate behaviors. This may be considered in light of the shyness that is characteristic of the 22q11DS phenotype: children but also adolescents and adults with 22q11DS are typically described as socially withdrawn and lacking initiative [21, 35]. Incidentally, the other distinction between the two clinical groups appeared in assertiveness (persistence/submission scale): participants with 22q11DS were less assertive than those with ASD. In line with this lack of initiation, it was observed that participants with 22q11DS were more passive than participants with ASD during the role-play, accepting more quickly the examiner’s refusal. Previous research has shown that peer-mediated interventions have beneficial effects on the ability to be engaged, participative and assertive during conversations (e.g., [80]). Moreover, training assertiveness was also found to have a positive impact on adolescents experiencing bullying by increasing courage, social communication satisfaction and competence in case of conflicts with peers (e.g., [81]). The use of such programs should be considered in individuals with 22q11DS in the school setting.

In addition to the observed quantitative differences in terms of social adequacy and assertiveness, qualitative differences between individuals with 22q11DS and ASD on some subscales were also noted by the examiners (even if the scores were statistically comparable between the two groups). This was driven by the fact that two participants could receive the same score on a given subscale but for different reasons. For example, one participant might receive a score of 3 in verbal fluency due to excessive pauses and initiation deficits, while another participant might receive a score of 3 due to repetitive speech and accelerated verbal flow. Another example would be non-verbal communication, where participants with 22q11DS most often exhibited lack of enthusiasm and warmth, whereas participants with ASD were also penalized on this subscale due to repetitive and stereotyped movements and lack of eye contact. These qualitative nuances are not reflected in the scores but in the descriptions to assign the scores, which justifies the need to develop more fine-grained subscales. In addition, (semi)-automated analyses of the discourse/social interaction could be used in future studies to obtain a more precise characterization of the socio-communicative profile in these two populations (e.g., [82]).

The post-hoc analyses have highlighted several characteristics of participants that are associated with social skills. First, social skills were significantly associated with IQ within each group. Indeed, lower IQ was associated with poorer SSPA performance in both participants with 22q11DS and with ASD. These findings are consistent with Bauminger et al. [83] who reported that children with ASD with lower IQ were less socially involved with their peers. In addition, spontaneous initiations were observed to be particularly difficult in unstructured activities such as leisure time [83]. In the present study, both structured (role-play 2) and less structured (role-play 1) situations were more difficult for participants with ASD and 22q11DS compared to TD, showing that individuals with cognitive impairments need additional support to develop their social skills. Second, while age did not appear to be associated with social skills, gender was. Indeed, females outperformed males in the TD and 22q11DS groups. The higher performance of the females could be explained by studies showing that females are more socially oriented than males in the general population (e.g., [84]), although gender differences are still debated. However, there was no difference between males and females in the ASD group. This result was surprising, given that the female profile of ASD has received increased attention recently and that studies indicate a less severe symptomatic profile than in males, including fewer social difficulties (e.g., [85–88]). However, it has also been reported that females with ASD experience increased socio-communicative impairments during adolescence compared to males (e.g., [89, 90]), which may explain why no differences were observed in our sample. In addition, females with ASD in our sample had a fairly high symptom severity score (females: *m* = 6, *sd* = 2.646; males: *m* = 8, *sd* = 1.864), suggesting that the autism symptomatology was fairly similar between males and females in our sample. Of note, our results also showed that the severity of autism symptoms were associated with social skills impairments, which emphasizes that social skills are an area of weakness and need to be addressed, especially in people with more severe symptoms. Furthermore, the "camouflage" hypothesis posits that women with ASD exhibit superficial social skills that help them mask their ASD symptoms [85, 86]. However, this is a mechanism that operates on the surface (e.g., [91]) and subtle impairments could be still visible through role-playing, thus possibly explaining the lack of differences between males and females. It should be noted that specific social skills training programs have been proposed for adolescent females with ASD (e.g., [92]), as most of the literature in ASD focuses on males.

#### Direct observation vs. caregiver report

While social skills appeared to be impaired in individuals with ASD and 22q11DS compared to TD, both as measured through direct observation (*i.e.* SSPPA) and as reported by parents (*i.e.* ERSSQ), the two measures were not associated with each other, showing low convergent validity. Additionally, a significant association between social skills and social functioning was observed in both clinical groups, but only when assessed through parent-report. On the other hand and in line with the previous findings [48, 55], the SSPA was not associated with social functioning. This raises the question of what is actually being measured by these tools, and the need for a combination of methods to fully capture complex constructs such as social skills. It is a common phenomenon across populations and fields that multi-method designs show poor associations between different measures, such as self- versus parent- or clinician-rated questionnaires [93–96].

Several arguments could be put forward to explain this lack of association. First, we believe there is a context effect when social skills are measured. Indeed, direct observation of social skills, as it is the case with the SSPA, takes place in specific contexts (i.e., meeting a new neighbor and retrieving a notebook). It is therefore information on social skills *in the moment* that may be different in another context such as meeting a friend or asking for something in a supermarket. On the contrary, the ERSSQ measures broader social skills that are not context-dependent, such as how well participants engage in general social behaviors like successfully handling social problems or initiating a conversation appropriately [69]. Second, the ERSSQ measures the frequency of exposure to social situations but does not provide information about how these social situations are experienced. Therefore, it would be interesting to add a rating scale to the ERSSQ to reflect the subjective aspect (*i.e.* how well) of social skills and not only the objective aspect (*i.e.* how often), as it is done in other area of social functioning, such as social anxiety.

### Aim 2: Does social anxiety play a role in social skills ?

Consistent with our hypotheses, social anxiety as measured by the SIAS was more prevalent in the clinical groups than in TD, and even more prevalent in individuals with ASD than with 22q11DS. Furthermore, this coincides with the percentage of individuals in our clinical groups meeting the formal criteria for a comorbid diagnosis of social anxiety (i.e., 2% of individuals with 22q11DS and almost 20% of individuals with ASD). However, contrary to our hypothesis, the self-reported measure of social anxiety was not associated with directly observed social skills, and it was only associated with parent-reported social skills in participants with 22q11DS. There are several possible explanations to explain these mixed results. First, it is still debated in the literature whether social skills and social anxiety are associated (e.g., [97]), and further studies are needed to disentangle the two mechanisms. Second, consistent with the present findings, there are studies that reported an association between social anxiety and adaptive functioning (including social features) in individuals with 22q11DS (e.g., [98]). This association is also reported in some studies in individuals with ASD (e.g., [99]), but others have reported weak correlations between social anxiety and social skills (e.g., [100]), indicating an unclear link. The lack of association in ASD participants in the present study could stem from the camouflage hypothesis discussed earlier: as ASD individuals may mask their difficulties, it could be more difficult for their parents to assess social skills. Lastly, this raises the question of the potential directionality of this association. Interestingly, a study showed that difficulties in communication and social interactions were associated with greater social anxiety later in life, but that the reverse relationship was much weaker (e.g., [41]). In the present study, only cross-sectional correlations were conducted so it would be relevant to examine the longitudinal associations between social skills and social anxiety in future studies.

### Strengths, clinical implications, limitations and future directions

The current study is in line with previous evidence and highlights that social skills are a specific area of weakness in youths with neurodevelopmental disorders. Since social skills have an impact on overall social functioning, and by cascade effect on self-esteem and well-being [3], it appears even more crucial to promote their development. In addition, the use of direct observation (i.e., role-plays) allowed us to capture for the first time the specific social skills profile of individuals with 22q11DS and ASD, highlighting the need for population-specific interventions. In this context, the SSPA could represent a useful tool for the assessment of social skills and provide a sufficiently fine-grained assessment to capture specific areas of strengths and weaknesses. Specifically, it could be used as a pre-intervention baseline to define the specific objectives and then reused at a later stage to examine the effectiveness of the intervention. Currently, the ADOS is often used in clinical practice to assess the severity of current symptoms, including social skills. However, the ADOS is an expensive diagnostic tool that requires extensive training and does not provide a fine-grained description of social skills. In addition, it also covers the area of restricted interests and repetitive behaviors that may not be specifically requested for reassessment. Thus, the SSPA could represent a more accessible alternative for the assessment of social skills.

It should be noted that the current study has several methodological limitations. First, the scoring of the role-plays relies on the subjective evaluations of the examiners, which may introduce biases. To overcome this limitation, we have put a particular effort in double scoring almost all the videos (96%). Then, although role-plays mimic real-world scenarios, there is no guarantee that participants would actually engage in these social interactions in real-world Morrison et al. [47].

Secondly, heterogeneity within the 22q11DS and ASD groups should be considered. First, a variety of comorbidities and medications were present in both clinical groups, which may have an impact on the results. However, comorbidities are the rule rather than the exception in neurodevelopmental disorders (e.g., [13, 101]), which is why the presence of comorbid psychiatric conditions was not defined as an exclusion criterion. In the 22q11DS group, potential comorbidity with ASD was not systematically investigated in depth, despite the presence of some participants with scores above the clinical cutoff on the SCQ. This is a limitation that will be addressed in future studies by conducting an ADOS assessment when the SCQ score is above the cutoff to allow comparison between 22q11DS participants without ASD and 22q11DS with comorbid ASD. However, the results were replicated without the participants with 22q11DS (*n* = 14) who scored above the clinical cutoff. We also point out that, among participants with 22q11DS, there was no difference in terms of social skills (SSPA and ERSSQ), regardless of their SCQ score. In other words, youths scoring above the clinical threshold on this questionnaire measuring difficulties in communication and social interaction were not characterized by specific social skills impairments compared to those scoring below the clinical threshold. This is important to consider as it indicates that social skills impairments are relatively independent of a possible comorbid diagnosis of ASD, but rather represent an intrinsic characteristic of this syndrome. Second, only two individuals with ASD had an IQ score in the intellectual disability range, indicating that these results cannot be extended to the full autism spectrum. Third, females represented 44% of our sample of autistic youths, which may not be representative of the classical male-to-female ratio reported in the literature, although this ratio is the subject of debate (e.g., [102]). The high percentage of females in our study may be explained by the fact that some of our participants were diagnosed late (i.e., not early childhood diagnosis), yet it has been shown in the literature that the age of diagnosis is frequently later in young women [103, 104]. Moreover, since the male prevalence and the male-to-female ratio was found to be lower in adults than in children with ASD (e.g., [105]), it could be another explanation for our high female ratio since we focused on the adolescence-early adulthood phase. Our male-to-female ratio should therefore be borne in mind when interpreting the results. Finally, due to the relatively verbally and cognitively demanding nature of the task, the present results cannot be extended to individuals with lower verbal and/or intellectual abilities.

## Conclusions

This study provides insight into the characterization of the social skills profiles of two populations often considered to share the same type of difficulties and highlights the need to develop interventions that specifically addresses the unique needs of each population. Moreover, the use of a methodology with high ecological validity (*i.e.* role-plays) allows the results to be generalized to everyday life, taking into account the limitations mentioned above. This study also shows the importance of combining both direct observation and parent-report measures, as these methods do not necessarily provide convergent results.

### Supplementary Information

**Supplementary Material 1.**

## Data Availability

The data set is publicly available through the YARETA data preservation system.
