# Supplementary material for: Social skills in neurodevelopmental disorders: a study using role-plays to assess adolescents and young adults with 22q11.2 deletion syndrome and autism spectrum disorders
Source: J Neurodev Disord. 2024 Mar 18;16:11. doi: 10.1186/s11689-024-09527-y (PMC11064408; doi:10.1186/s11689-024-09527-y)
Supplement: Supplementary file 1 — Supplementary Material 1. [file 11689_2024_9527_MOESM1_ESM.docx]

**Social skills with with IQ, gender and age as covariates**

As non-parametric tests were conducted, covariates could not be added in the models. To account for potential confounding variables, we ran a MANOVA as post-hoc analysis with a control of gender, age and IQ. By doing so, social skills differences between TD and clinical groups remain similar (TD-22q11DS: *F*(11) = 2.606, *p* = .006, η^2^ = .230; TD-ASD: *F*(10) = 11.665, *p* = < .001, η^2^ = .599). Moreover, distinct profile emerged between ASD and 22q11DS participants (*F*(11) = 3.309, *p* < .001, η^2^ = .342).

**Social skills role-plays 1 and 2**

When comparing role-play 1 and 2, similar results were found than in SSPA total score that is made of role-play 1 and 2. Statistically significant differences were observed among the groups for role-play 1 (*H*(2) = 70.932, *p* < .001, η^2^ = 0.466) and role-play 2 (*H*(2) = 68.272, *p* < .001, η^2^ = 0.448). In both role-plays, post-hoc analysis showed that TD participants showed higher social skills performances than both participants with 22q11DS ((role-play 1: (*H*(2) = 53.901, *p* < .001, η^2^ = 0.46); role-play 2: (*H*(2) = 59.491, *p* < .001, η^2^ = 0.509) and with ASD (role-play 1: (*H*(2) = 68.297, *p* < .001, η^2^ = 0.701); role-play 2: (*H*(2) = 58.573, *p* < .001, η^2^ = 0.6)). There was no difference between the clinical groups (role-plays 1 and 2: *p* > 0.05).

**SSPA subscales performance with IQ, gender and age as covariates**

|  | 22q11DS-ASD | | |
| --- | --- | --- | --- |
|  | Statistical test | *p*-value | η*2* |
| SSPA total non-verbal communication and affect (RP 1+2) | 4.031 | **.048** | .048 |
| SSPA total clarity (RP 1+2) | 20.396 | **.000** | .203 |
| SSPA total fluency (RP 1 + 2) | 10.306 | **.002** | .114 |
| SSPA total focus (RP 1 + 2) | 3.891 | .052 | .046 |
| SSPA total involvement (RP 1 + 2) | 6.001 | **.016** | .070 |
| SSPA total social adequacy (RP 1+2) | 5.980 | **.017** | .070 |
| SSPA overall conversation (RP 1) | 5.464 | **.022** | .064 |
| SSPA overall argument (RP 2) | .347 | .558 | .004 |
| SSPA negociation ability (RP 2) | 1.942 | .167 | .024 |
| SSPA submission/persistence (RP 2) | 4.711 | **.033** | .056 |

*Significant p-values at the 0.05 level are displayed in bold*

**Additional analyses**

To further explore the association between social skills and general characteristics, the associations with IQ, age, genre and ASD symptomatology were explored.

*a. IQ:* There was no association between social skills and IQ in TD participants (with SSPA total score: *r* = .041, *p* = .755; with ERSSQ total score: *r* = .034, *p* = .809). However, in participants with 22q11DS, higher IQ was significantly associated with higher social skills, regardless of the assessment tool (SSPA total score: *r* = .314, *p* = .024; ERSSQ total score: *r* = .429, *p* < .001). In participants with ASD, the association with IQ was significant only with the SSPA total score (*r* = .407, *p* = .017) but not with the ERSSQ total score (*r* = -.233, *p* = .191).

*b. Age:* There was no association between the SSPA total score and age or between the ERSSQ total score and age (all *p* > 0.05; data not shown).

*c. Gender:* In the entire sample, statistically significant gender differences were observed in terms of social skills as measured with the SSPA, with higher social skills being observed in females compared to males (*H*(1) = 10.318, *p* < .001, η^2^ = 0.063). *Post-hoc* intra-group analyses showed that females showed higher social skills performances than males both in TD (*H*(1) = 4.083, *p* = .043, η^2^ = 0.065) and 22q11DS groups (*H*(1) = 8.279, *p* = .004, η^2^ = 0.159). However, there was no gender difference in the ASD group (*H*(1) = .784, *p* = .376, η^2^ = 0.024). *Post-hoc* inter-group analyses conducted in males and females separately showed similar results than in the overall group (see section 3.2). On the opposite, there was no statistically significant gender differences when social skills were measured with the ERSSQ (*H*(1) = 2.242, *p* = .134, η^2^ = 0.002). Moreover, *post-hoc* inter-group analyses conducted in males and females separately showed similar results than in the overall group (see section 3.2).

*d. ASD symptomatology:* In participants with ASD, the ADOS severity score was significantly associated with the SSPA total score (*r* = -.519, *p* = .002) but not with the ERSSQ total score (*r* = .113, *p* = .530).

**Comparisons between participants with 22q11DS scoring below and above the clinical cutoff on the SCQ**

Participants with 22q11DS scoring below (N=39) and above (N=14) the clinical cutoff on the SCQ did not differ statistically in terms of social skills, both when assessed with role-plays (SSPA: U = 270.5, *p* = .689) and as reported by caregivers (ERSSQ: U = 178.5, *p* = .112).
